# Analysis of Postural Control Using Principal Component Analysis: The Relevance of Postural Accelerations and of Their Frequency Dependency for Selecting the Number of Movement Components

**DOI:** 10.3389/fbioe.2020.00480

**Published:** 2020-05-19

**Authors:** Arunee Promsri, Peter Federolf

**Affiliations:** ^1^Department of Sport Science, University of Innsbruck, Innsbruck, Austria; ^2^Department of Physical Therapy, University of Phayao, Phayao, Thailand

**Keywords:** movement strategy, neuromuscular control, filtering, frequency analysis, principal component analysis PCA, principal acceleration

## Abstract

One criterion when selecting the number of principal components (PCs) to be considered in a principal component analysis (PCA) is the fraction of overall variance that each PC represents. When applying a PCA to kinematic marker data in postural control research, this criterion relates to the amplitude of postural changes, recently often called “principal (postural) positions” (PPs). However, in the assessment of postural control, important aspects are also how fast posture changes and the acceleration of postural changes, i.e., “principal accelerations” (PAs). The current study compared how much of the total position variance each PP explained (PP_rVAR) and how much of the total acceleration variance each PA explained (PA_rVAR). Furthermore, the frequency content of PP and PA signals were evaluated. Postural movements of 26 participants standing on stable ground or balancing on a multiaxial balance board were analyzed by applying a PCA on 90 marker coordinates. For each PC, PP_rVAR, PA_rVAR, and the Fourier transformations of the PP and PA time series were calculated. The PP_rVAR and the PA_rVAR-distributions differed substantially. The PP-frequency domain was observed well below 5 Hz, the PA-frequency domain up to 5 Hz for stable standing and up to 10 Hz on the balance board. These results confirm that small-amplitude but fast movement components can have a higher impact on postural accelerations—and thus on the forces active in the system—than large-amplitude but slow lower-order movement components. Thus, PA variance and its dependence on filter frequencies should be considered in dimensionality reduction decisions.

## Introduction

Principal component analysis (PCA) is an unsupervised data analysis procedure often used as a preprocessing step, e.g., to improve performance or for dimensionality reduction, before more complex machine learning procedures are applied. If applied in the analysis of human motion, a PCA can by itself reveal interesting information about the coordinative structure of complex whole-body movements. Accordingly, applying a PCA on kinematic data has received increasing attention in research on several kinds of human movements, such as reaching (Longo et al., 2019), karate kicking (Zago et al., 2017a), juggling (Zago et al., 2017b), skiing (Federolf et al., 2014; Gløersen et al., 2018; Pellegrini et al., 2018), or walking (Troje, 2002; Daffertshofer et al., 2004; Verrel et al., 2009; Zago et al., 2017c).

One of the main purposes for performing a PCA on kinematic data—or in fact on any dataset—is the idea that the entire variance in the data can often be approximated to high accuracy with only a limited number of principal components (PCs). One of the most common criteria for choosing the number of PCs is the eigenvalue spectrum, which represents the variance explained by each PC and which can be expressed in relative values, i.e., as a percentage of the entire variance in the data.

A research area where PCA has been particularly frequently applied on kinematic human movement data is research on postural control (Federolf et al., 2013; Federolf, 2016; Haid and Federolf, 2018; Haid et al., 2018, 2019; Promsri et al., 2018, 2019, 2020a,b; Wachholz et al., 2019a,b). In postural control studies, when PCA is applied to kinematic data, it decomposes the complex multi-segment whole-body movements into a set of one-dimensional movement components, called “principal movements” PM_k_, where k is the order of the movement component (Federolf et al., 2013; Federolf, 2016). Previous research has shown that the lower-order PM_k_ represent in close approximation the classical motor strategies (Horak and Nashner, 1986; Winter, 1995), i.e., the ankle or hip strategies (Federolf, 2013). If PCA is calculated on normalized data from different volunteers, then a subject-specific *relative explained variance* can be calculated in analogy to the eigenvalues, which quantify the explained variance for the whole dataset and are thus not subject-specific. The *relative explained variance-*spectra provide one criterion for how many movement components PM_k_ one wants to consider in the analysis (Federolf, 2013; Haid et al., 2019).

However, analyzing the different postures observed during a measurement sequence may not be the only variable of interest. How fast the posture changes and how much a postural change is accelerated, also provide valuable information. We have shown in previous papers, that Newton's mechanics can be applied to the PCA-based posture space by defining a “principal (postural) position” (PP_k_) for each PM and their time derivatives, principal velocity (PV_k_) and principal acceleration (PA_k_) (Federolf, 2016; Longo et al., 2019). The PA_k_ are of interest, since they relate to forces acting in the system and thus to the neuromuscular control of the postural movements (Federolf, 2016; Haid et al., 2018; Promsri et al., 2018, 2019, 2020a,b; Haid and Federolf, 2019; Wachholz et al., 2019a,b, 2020). We want to emphasize here that the PA_k_ obtained by double-differentiation of the PP_k_ time series (Federolf, 2016; Longo et al., 2019) are different variables than when a PCA is performed directly on acceleration data (Verheul et al., 2019): The former can be seen as an expansion of the movement strategy concept (Horak and Nashner, 1986; Winter, 1995), since the PA_k_ quantify the acceleration of the considered movement components/movement strategies; the latter PCA identifies correlated patterns directly in acceleration data, which yields a different solution.

Differentiation is a non-linear operation and, consequently, the relative variance spectra of the PP_k_ differ from the PA_k_ relative variance spectra (Longo et al., 2019). Particularly in postural control it is likely that large-amplitude, yet slow movement components influence the PA-spectrum less than small-amplitude, but fast movement components. The PA-explained variance spectrum could be a second important criterion for the decision on how many PM_k_ should be considered in an analysis (Longo et al., 2019). Unfortunately, noise amplification in differentiation makes a filtering of the PP_k_ signals necessary before PV_k_ and PA_k_ are calculated (Winter et al., 1974), and since the PA_k_ variance spectra are speed-dependent, they will change with the filter cut-off frequency used before the differentiation.

In summary, when applying a PCA to investigate the coordinative structure of postural control movements, both the principal positions (PP_k_) and the principal accelerations (PA_k_) are of interest since they provide relevant information on the composition of the postural movements and on the control of the movement components, respectively (Promsri et al., 2020a). Both the PP_k_- and PA_k_-spectra should be considered when selecting the number of movement components to be analyzed, however, the PA_k_-spectra are speed- and thus filter frequency-dependent. Thus, the purposes of the current Brief Research Report were (i) to compare the PP_k_ and PA_k_ relative variance spectra for postural control data; (ii) to evaluate the frequency content of the PP_k_ and PA_k_ time series; and (iii) to assess how the PA relative variance spectrum depends on the filtering cut-off frequency.

## Materials and Methods

### Participants

Twenty-six physically active young adults (14/12 males/females, age 25.3 ± 4.2 years, weight 70.7 ± 11.4 kg, height 175.0 ± 8.1 cm, physical activity participation 8.4 ± 5.4 h/weeks [mean ± SD]) with no neuromuscular injuries/disorders and no specific balance training participated in the current study. All volunteers provided informed consent and the study protocol had been approved by the Board of Ethical Questions in Science of the University of Innsbruck, Austria (Certificate 16/2016).

### Measurement Procedures

Participants were equipped with 39 reflective-markers according to the “Plug-In Gait” marker setup (Vicon Motion Systems Ltd., Oxford, UK). Two 80-s barefooted-bipedal balancing trials, one for each support surface, were completed in randomized order on a firm surface (FS) and on a wobble board (WB; Powrx Balance Board; POWRX GmbH., Germany). After completing the first trial, participants could rest for up to 3 min. For the WB condition, volunteers had a 15-s familiarization trial with no instruction or feedback. Postural movement trajectories were captured by a standard 8-camera motion tracking system (Vicon Bonita B10 cameras with Nexus 2.2.3 software; Vicon Motion Systems Ltd., Oxford, UK) using a sampling rate of 250 Hz.

To standardize the standing position (Supplementary Figure 1), participants were asked to place two marked points (base of each 2nd metatarsal bone) over a horizontal line taped on the floor for FS or over a horizontal diameter of the WB; to align the inside of the feet (the medial borders of each distal end of the first metatarsal bone) with tapes defining an individual inter-feet distance (15% of biacromial diameter); to rest their hands on the hips; and to look straight ahead at a 10-cm-diameter red-circle target on a wall at the individual eye level ~5 m away. To standardize the position of the wobble board, we placed the center of the wobble board over the center of a reticle cross-line marked on the floor. During testing, volunteers were asked to stand still for the FS or to keep the board horizontal for the WB; to avoid any voluntary movements; and to keep their eyes on the target.

### Data Analysis

#### Kinematic Data Pre-processing

All data processing was conducted in Matlab (MathWorks Inc., Natrick, MA, USA). The pre-processing steps and the PCA analysis were conducted based on earlier studies (Federolf, 2016; Promsri et al., 2018, 2019, 2020a). Briefly, any gaps in marker trajectories were filled by a PCA-based reconstruction technique (Federolf, 2013; Gløersen and Federolf, 2016). Two PCAs were performed, one for each balancing condition (Promsri et al., 2020a). The middle 60 seconds of each balancing trial were extracted and nine asymmetrical markers placed on the upper arms, lower arms, right scapular, upper thighs, and the lower thighs were omitted. In analogy to previous studies (Troje, 2002; Daffertshofer et al., 2004; Verrel et al., 2009; Federolf, 2016), the 3D coordinates (x, y, z) of the remaining 30 markers of each dataset at a given time *t* were interpreted as 90-dimensional posture vectors:

(1) $$\begin{array}{l} \vec{p\left(t\right)}=\left[x_{1}\left(t\right)\;,\;y_{1}\left(t\right),\;z_{1}\left(t\right),\ldots ,x_{30}\left(t\right),\;y_{30}\left(t\right),\;z_{30}\left(t\right)\right] \end{array}$$

Three pre-processing steps were then conducted. First, the posture vectors were centered by subtracting the subject's mean posture vector. For each subject, *subj*, a mean posture vector:

(2) $$\begin{array}{l} \vec{\bar{p^{subj}}}=\left[x_{1}\;,\;y_{1},\ldots ,\;z_{30}\right] \end{array}$$

where the bar over the variable indicates the mean over time, $x=mean_{t}\left(x\left(t\right)\right)$, was subtracted from each posture vector:

(3) $$\begin{array}{l} \vec{p^{\prime }\left(t\right)}=\vec{p\left(t\right)}-\;\vec{\bar{p^{subj}}} \end{array}$$

This procedure is the first step toward removing anthropometric differences (Federolf, 2016). The PCA was, therefore, conducted on deviations from a subject's mean posture, i.e., on postural movements. Second, the centered posture vectors were normalized to the mean Euclidean distance $d^{subj}$ (Federolf, 2013, 2016). Thus, for each posture vector $\vec{p^{\prime }\left(t\right)}$ the Euclidean norm:

(4) $$\begin{array}{l} d^{subj}\left(t\right)=\left(\sqrt{{x_{1}\left(t\right)}^{2}+{y_{1}\left(t\right)}^{2}+{z_{1}\left(t\right)}^{2}+\ldots +{z_{30}\left(t\right)}^{2}}\right) \end{array}$$

was calculated and the $\vec{p^{\prime }\left(t\right)}$ were then divided by the mean of these Euclidian distances:

(5) $$\begin{array}{l} \vec{p^{\prime \prime }\left(t\right)}=\frac{1}{\bar{d^{subj}}}\;\vec{p^{\prime }\left(t\right)}. \end{array}$$

Third, the normalized posture vectors were weighted using sex-specific mass distributions (Gløersen et al., 2018). Specifically, for each marker *i* a weight factor *w*_*i*_ was calculated by dividing the relative weight of the segment to which the marker was attached, *m*_s_, by the number *n*_*s*_ of markers on this segment. For markers placed on joints, the masses of both segments were added. For example, *w*_*i*_ for the knee markers was calculated as $w_{i}=\frac{m_{thigh}}{n_{thigh}}+\;\frac{m_{shin}}{n_{shin}}$ with *n*_*thigh*_ = *n*_*shin*_ = 3, *m*_*thigh*_ = 14.16%, and *m*_*shin*_ = 4.33% for men (de Leva, 1996). Thus, the normalized postural movement vectors had the form:

(6) $$\begin{array}{l} \vec{p^{\prime \prime \prime }\left(t\right)}=\frac{1}{\bar{d^{subj}}}\left[w_{1}\left(x_{1}\left(t\right)-\;\bar{{x_{1}}^{subj}}\;\right),\;w_{1}\left(y_{1}\left(t\right)-\;\bar{{y_{1}}^{subj}}\;\right),\right]\\ \;\left[\ldots ,w_{30}\left(z_{30}\left(t\right)-\;\bar{{z_{30}}^{subj}}\;\right)\right] \end{array}$$

Then, the normalized posture vectors $\vec{p^{\prime \prime \prime }\left(t\right)}$ from all volunteers were concatenated to form a 390,000 × 90-PCA input-matrix (250 [sampling rate] ^*^ 60 [trial duration] ^*^ 26 [number of subjects] × 90 [marker coordinates]).

#### Principal Component Analysis

The PCA was calculated by a singular-value decomposition of the input matrix's covariance matrix and produced a set of PC-eigenvectors, $\vec{{\text{P}\text{C}}_{\text{k}}}$, which form a new basis for the vector space of marker positions (Haid et al., 2019). All PC-eigenvectors are linear combinations of the original marker coordinates. Animated stick figures can be created from the mean postures and from each eigenvector to characterize the principal movements PM_k_ (Federolf et al., 2013; Federolf, 2016). The time evolution of each PM_k_, i.e., the PP_k_(t), were obtained by a coordinate transformation of the normalized posture vectors onto the PCA-eigenvectors.

$$\begin{array}{l} PP_{k}\left(t\right)=\vec{p^{\prime \prime \prime }\left(t\right)}\;\cdot \;\vec{PC_{k}} \end{array}$$

The PP_k_(t) represent positions in posture space, i.e., how much the posture at time *t* deviates in the direction of the PC_k_-eigenvector from the mean posture (Federolf, 2016). In other words, the PP_k_(t) represent the amplitude of each movement component PM_k_. The variance of each PP_k_(t), divided by the sum of the variances of all PP_k_(t), results in a variable *relative explained variance of principal position PP*_*rVAR*_k_ that quantifies for each volunteer and each order k, how much the specific PM_k_ contributed to the whole postural movements of the subject.

In analogy to Newton's mechanics and differentiation rules, the rate of postural change can be quantified by principal velocities PV_k_(t), i.e., by the first time derivative of the PP_k_(t), $\text{P}{\text{V}}_{\text{k}}=\frac{\text{d}}{\text{d}\text{t}}\;\text{P}{\text{P}}_{\text{k}}$; and the acceleration of postural movements can be quantified by principal accelerations PA_k_(t), i.e., by the second time derivative of the PP_k_(t), $\text{P}{\text{A}}_{\text{k}}=\frac{{\text{d}}^{2}}{\text{d}{\text{t}}^{2}}\;\text{P}{\text{P}}_{\text{k}}$ (Federolf, 2016). In case of unperturbed human postural control, PA_k_(t) are either a direct result of muscle activation, a result of the neuromuscular system utilizing gravity to produce desired accelerations, or an undesired result of gravity which the neuromuscular system was not able to prevent e.g., loss of stability (Promsri et al., 2020a). In this sense, the PA_k_(t) are the essential mechanical variables that the sensorimotor system must control in order to govern the body's motion and maintain its stability. Thus, each PA_k_(t) represents a variable that quantifies how the mechanical system is controlled (Federolf, 2016; Promsri et al., 2020a). In analogy to *PP*_*rVAR*_k_, we calculated the variable *relative explained variance of principal acceleration PA_rVAR*_*k*_ to assess how much each movement component contributed to the overall postural accelerations in the individual subjects.

Due to noise amplification in the differentiation processes (Winter et al., 1974), filtering of the PP_k_(t) is needed before computing PV_k_(t) and PA_k_(t). The current study examined the effect of low-pass filtering using a 3rd-order, zero-phase, low-pass Butterworth filter. The Butterworth filter was selected, since it is free of ripples in the pass and stop band. The filter order (3rd) was selected arbitrarily, however, preliminary tests suggested that the filter order has a very small effect on the PA time series. Prior to filtering, the frequency contents of the raw PP_k_(t) and PA_k_(t) were evaluated using a Fourier transformation. Then, the effect of cut-off frequency on *PA_rVAR*_*k*_ was evaluated for both balancing situations, FS and WB, with cut-off frequencies of 1, 2, 5, 10, and 20 Hz and with no filtering. Finally, explained variance spectra of *PP_rVAR*_*k*_ and *PA_rVAR*_*k*_ (10 Hz) were compared.

## Results

**Figure 1:**
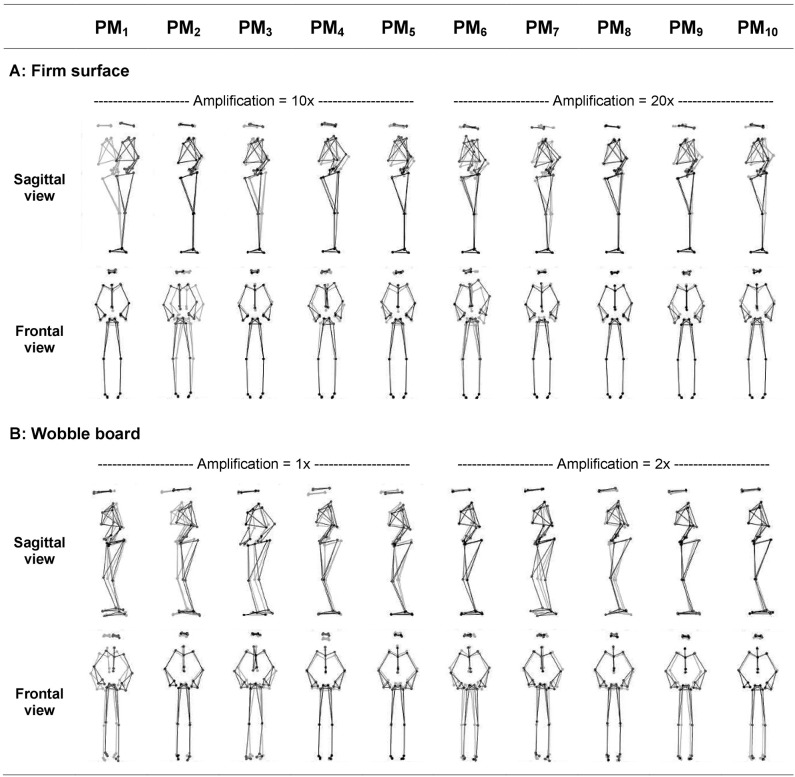
Illustration of the first ten principal movements (PM_1−10_) of bipedal standing on (**A)** the firm surface and bipedal balancing on **(B)** the wobble board. Gray and black lines/dots show the extreme posture in opposite directions. Movement amplitudes are amplified using the indicated factor for a better visualization (Firm surface: amplification 10× for PM_1−5_, and 20× for PM_6−10_; Wobble board: amplification 1× for PM_1−5_, and 2× for PM_6−10_). Movements are clearer and can be more easily characterized when viewed in animated stick figure videos: Supplementary Videos S1, S2 for balancing on the firm and soft surfaces, respectively.

**Figure 2:**
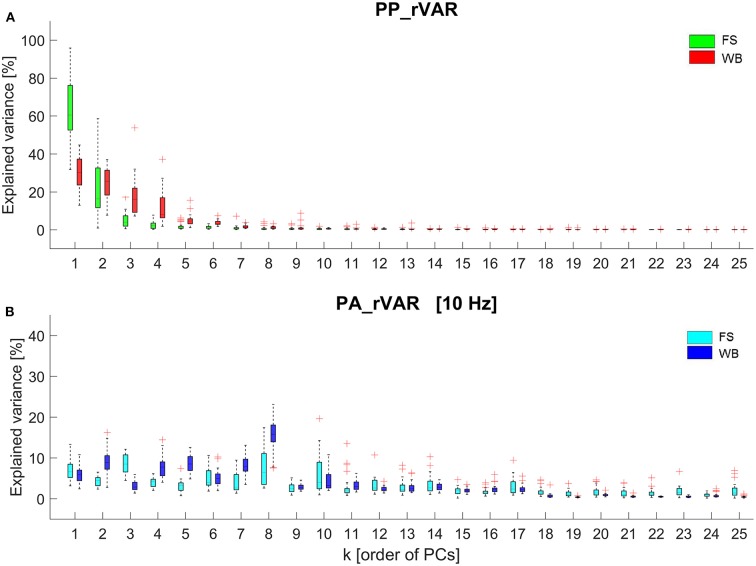
Box plots representing the data from all 26 participants of **(A)** the relative explained variance of principal postural positions (*PP_rVAR*_k_) and **(B)** the relative explained variance of principal postural accelerations (*PA_rVAR*_k_) of standing on the firm surface (FS) and balancing on the wobble board (WB). The *PA_rVAR*_k_ were determined after filtering the data with a 3rd-order 10 Hz low-pass Butterworth filter.

The first 10 principal movements (PM_1−10_) of standing on a firm surface (FS) and balancing on a wobble board (WB) are described and shown in (Table 1, Figure 1), and in (Supplementary Videos 1, 2). Higher-order movement components were not included for the visualization and description, since their small movement amplitudes make them difficult to characterize, however, higher-order components were considered in the evaluation of the variance spectra. The spectra of explained variance, *PP_rVAR*_*k*_ and *PA_rVAR*_*k*_ (for a cut-off frequency of 10 Hz) are shown in (Figure 2). As expected, several movement components that contributed little to the postural variance did have an over-proportional contribution to the acceleration variance. Specifically, for standing on the FS, PM_3_, PM_8_, and PM_10_ which predominantly represented hip strategy and upper body movements, and for balancing on the WB PM_8_ which predominantly quantified ankle plantar/dorsiflexion, were of particular interest.

**Table 1:** The relative explained variance of principal position *PP_rVAR* (%) and a qualitative description of the movement patterns represented by the first ten principal movements (PM_1−10_).

**PM**	***PP_rVAR* (%)**	**Main movements**
**A: Firm surface**		
1	64.4 ± 16.3	Anteroposterior ankle strategy
2	19.9 ± 13.5	Mediolateral ankle strategy (lateral weight shift)
3	4.8 ± 3.8	Anteroposterior hip strategy
4	2.3 ± 1.9	Transverse rotation of pelvis and upper body
5	1.8 ± 1.8	Vertical breathing movement patterns
6	1.3 ± 0.8	Vertical breathing movement patterns
7	1.0 ± 1.4	Anteroposterior trunk flexion coupled with knee flexion and extension
8	0.7 ± 1.0	Breathing; small chest movements
9	0.6 ± 0.7	Retraction and protraction of shoulders
10	0.4 ± 0.4	Upper body movement
**B: Wobble board**		
1	29.9 ± 8.5	Mediolateral ankle strategy (lateral weight shift)
2	24.2 ± 8.3	Anteroposterior ankle strategy
3	17.9 ± 10.5	Transverse; twisting of the board coupled with the whole-body rotation
4	11.7 ± 8.2	Anteroposterior hip strategy coupled with anteroposterior ankle strategy
5	5.0 ± 3.0	Vertical hip, knee, and ankle flexion/extension
6	3.7 ± 1.3	Mediolateral hip strategy coupled with mediolateral ankle strategy
7	1.6 ± 0.8	Diagonal lateral weight shift
8	1.1 ± 0.6	Anteroposterior ankle plantarflexion/dorsiflexion
9	1.1 ± 1.9	Twisting of the board coupled with the whole-body rotation
10	0.5 ± 0.3	Lateral weight shift and with small rotation

*Together, PM_1−10_ explained 97.2 and 96.7% of the overall postural variance when balancing on (**A)** firm surface, and **(B)** wobble board, respectively*.

**Figure 3:**
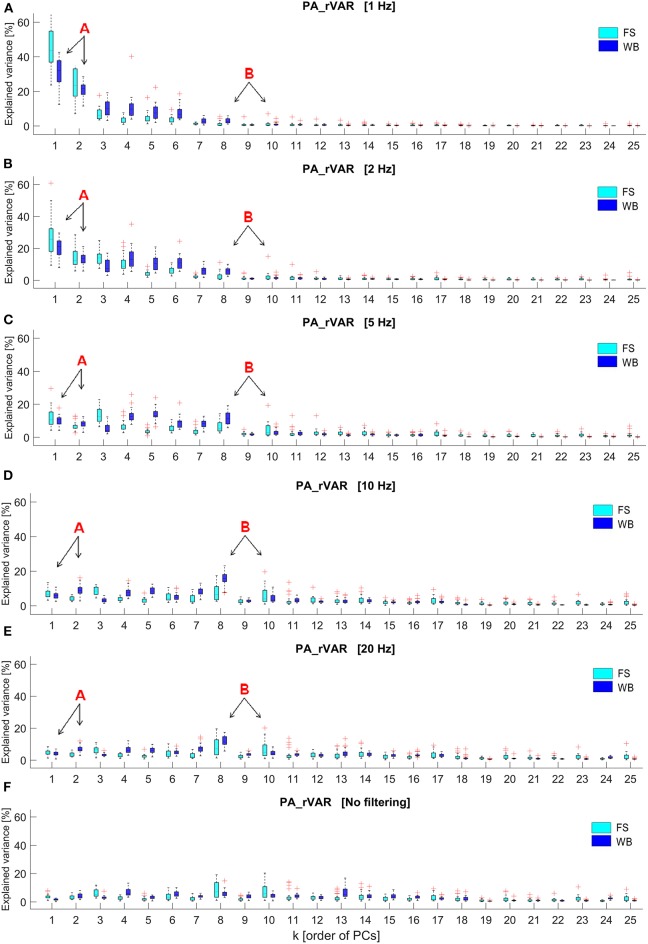
Box plots of the relative explained variance of principal postural acceleration (*PA_rVAR*_k_) of standing on the firm surface (FS) and balancing on the wobble board (WB) with different cut-off frequencies, including **(A)** 1 Hz, **(B)** 2 Hz, **(C)** 5 Hz, **(D)** 10 Hz, **(E)** 20 Hz, and **(F)** no filtering, which were observed from 26 participants (k displays order of principal components, PMs; k = 1 to 25). The letter, “A,” and its arrows point to lower-order PAs, *PA_rVAR*_1_, and *PA_rVAR*_2_, whose contribution to the overall acceleration variance decrease with increasing cutoff frequencies. The letter “B” and its arrows highlight two medium-order PAs, *PA_rVAR*_8_, and *PA_rVAR*_10_, whose contribution to the overall acceleration variance increase as cutoff frequencies are increased.

Fourier transformations of the raw PP and PA time series of one arbitrarily selected, representative volunteer are shown in (Supplementary Figure 2) for FS and in (Supplementary Figure 3) for WB. The PP-frequency domain of both FS and WB conditions was observed well below 5 Hz. In contrast, in the PA-spectra, despite the strong and blue-shifted noise, signals are visible in the ranges 0–5 Hz for FS and in the range up to ~10 Hz for WB. In addition, (Figure 3) illustrates how the spectrum of explained variance *PA_rVAR*_*k*_ changes with increasing filter cut-off frequency.

## Discussion

Our analysis demonstrates that PA_k_ and PA_k_-based variables, here *PA_rVAR*_*k*_, depend on the filter cut-off applied in the PA calculation. Low cut-off frequencies (<5 Hz) lead to over-pronunciation of slow movement components. As filter frequency is increased (5–20 Hz) a new pattern emerges, in line with the expectation that some of the higher-order movement components contribute more than other movement components to the accelerations. The Fourier analysis of the underlying signals suggests that the pattern emerging with increasing filter cut-offs is not (not only) a consequence of noise increasingly affecting the signal: while the PP_k_(t) live in a very low frequency range (<3 Hz), several of the PA_k_(t) show a relevant frequency content up to ~5 Hz in FS and up to 10 Hz in the WB conditions. These observations suggest that filter cut-off frequencies of 5–7 Hz for FS and around 10 Hz for WB would be appropriate.

The current findings underpin that (i) when focusing only on the classical movement strategies (lower-order PMs), one might overlook movement components that are small in posture-amplitude, but that can be accelerated fast and thus provide an important contribution to postural control. Spectra of PA-explained variance should be considered when deciding on how many PC-components are included in an analysis. (ii) When interested in neuromuscular control and thus in the accelerations and forces controlling postural movements, then filter frequencies should not be selected below 5 Hz for stable situations and not below 10 Hz for more dynamic balancing trials. The current findings corroborate the findings of Longo et al. (2019), who assessed PA-relative variance in a cyclic upper-body motion. Moreover, Longo et al. (2019) also mathematically validated that all PA_k_ together (i.e., the sum of all PA_k_) represent the entire marker accelerations present in the dataset. The current results also agree with previous studies in which the dependence of PA_k_ variables on filter cut-off frequencies was assessed, and which reported consistent results for cut-off frequencies in the range 5 to 12 Hz (Haid et al., 2018; Promsri et al., 2018, 2019, 2020a). Furthermore, recent studies on muscle synergies and on coherence between electromyographic signals from different muscles also reported spectra peaking around 9 Hz and posture-related coherence in frequency bands 5–20 Hz (Boonstra et al., 2008, 2015), which supports the assumption that the PA_k_ signals in this frequency range are of physiological origin and probably not an artifact or noise phenomenon.

The role of movement analysis in monitoring and diagnosing neurodegenerative conditions is increasingly recognized, particularly when combined with machine/deep learning approaches (Buckley et al., 2019). However, how successful such approaches can become depends largely on the information contained in the input data to these algorithms. Disregarding information at an early stage, e.g., due to dimensionality reduction or through filtering, is a form of investigator bias that likely affects even the performance of so-called unsupervised methods. Driven by biomechanical considerations, the current study evaluated what information might be contained in the often disregarded higher-order PC-components. The question, which specific PC_k_ components are relevant, depends on the specific movement, the specific boundary conditions that are present, and the research question that is studied. However, as general advise the current study suggest that *PA_rVAR*_*k*_-spectra should be analyzed when deciding on how many PC components are to be considered; and the frequency content and suitable filters should be carefully assessed in the calculation of PAs.

## Data Availability Statement

The datasets generated for this study are available on request to the corresponding author.

## Ethics Statement

The study reported here was approved by the Board of Ethical Questions in Science of the University of Innsbruck, Austria (Certificate 16/2016). All participants provided informed written consent prior to their participation.

## Author Contributions

AP and PF have contributed equally to the design and implementation of the research and to the writing of the manuscript.

## Conflict of Interest

The authors declare that the research was conducted in the absence of any commercial or financial relationships that could be construed as a potential conflict of interest.

## Supplementary Material

Supplementary Video 1 Principal movements of bipedal standing.

Supplementary Video 2 Principal movements of bipedal standing on a wobble board.

## References

[B1] Boonstra T. W. Daffertshofer A. van Ditshuizen J. C. van den Heuvel M. R. C. Hofman C. Willigenburg N. W.. (2008). Fatigue-related changes in motor-unit synchronization of quadriceps muscles within and across legs. J. Electromyogr. Kinesiol. 18, 717–731. 10.1016/j.jelekin.2007.03.005 17462912

[B2] Boonstra T. W. Danna-Dos-Santos A. Xie H. B. Roerdink M. Stins J. F. Breakspear M. (2015). Muscle networks: connectivity analysis of EMG activity during postural control. Sci. Rep. 5:17830. 10.1038/srep17830 26634293 PMC4669476

[B3] Buckley C. Alcock L. McArdle R. Ur Rehman R. Z. Del Din S. Mazzà C.. (2019). The role of movement analysis in diagnosing and monitoring neurodegenerative conditions: insights from gait and postural control. Brain Sci. 9:34. 10.3390/brainsci9020034 30736374 PMC6406749

[B4] Daffertshofer A. Lamoth C. J. C. Meijer O. G. Beek P. J. (2004). PCA in studying coordination and variability: a tutorial. Clin. Biomech. 19, 415–428. 10.1016/j.clinbiomech.2004.01.005 15109763

[B5] de Leva P. (1996). Adjustments to Zatsiorsky-Seluyanov's segment inertia parameters. J. Biomech. 29, 1223–1230. 10.1016/0021-9290(95)00178-6 8872282

[B6] Federolf P. Reid R. Gilgien M. Haugen P. Smith G. (2014). The application of principal component analysis to quantify technique in sports. Scand. J. Med. Sci. Sports 24, 491–499. 10.1111/j.1600-0838.2012.01455.x 22436088

[B7] Federolf P. Roos L. Nigg B. M. (2013). Analysis of the multi-segmental postural movement strategies utilized in bipedal, tandem and one-leg stance as quantified by a principal component decomposition of marker coordinates. J. Biomech. 46, 2626–2633. 10.1016/j.jbiomech.2013.08.008 24021753

[B8] Federolf P. A. (2013). A novel approach to solve the “missing marker problem” in marker-based motion analysis that exploits the segment coordination patterns in multi-limb motion data. PLoS ONE 8:e78689. 10.1371/journal.pone.0078689 24205295 PMC3813748

[B9] Federolf P. A. (2016). A novel approach to study human posture control: “principal movements” obtained from a principal component analysis of kinematic marker data. J. Biomech. 49, 364–370. 10.1016/j.jbiomech.2015.12.030 26768228

[B10] Gløersen Ø. Federolf P. (2016). Predicting missing marker trajectories in human motion data using marker intercorrelations. PLoS ONE 11:e0152616. 10.1371/journal.pone.0152616 27031243 PMC4816448

[B11] Gløersen Ø. Myklebust H. Hallén J. Federolf P. (2018). Technique analysis in elite athletes using principal component analysis. J. Sports Sci. 36, 229–237. 10.1080/02640414.2017.1298826 28287028

[B12] Haid T. Federolf P. (2018). Human postural control: assessment of two alternative interpretations of center of pressure sample entropy through a principal component factorization of whole-body kinematics. Entropy 20:30 10.3390/e20010030 PMC7512231 33265120

[B13] Haid T. Federolf P. (2019). The effect of cognitive resource competition due to dual-tasking on the irregularity and control of postural movement components. Entropy 21:70 10.3390/e21010070 PMC7514179 33266786

[B14] Haid T. H. Doix A.-C. M. Nigg B. M. Federolf P. A. (2018). Age effects in postural control analyzed via a principal component analysis of kinematic data and interpreted in relation to predictions of the optimal feedback control theory. Front. Aging Neurosci. 10:22. 10.3389/fnagi.2018.00022 29459826 PMC5807376

[B15] Haid T. H. Zago M. Promsri A. Doix A.-C. M. Federolf P. A. (2019). PManalyzer: a software facilitating the study of sensorimotor control of whole-body movements. Front. Neuroinform. 13:24. 10.3389/fninf.2019.00024 31024286 PMC6461015

[B16] Horak F. B. Nashner L. M. (1986). Central programming of postural movements: adaptation to altered support-surface configurations. J. Neurophysiol. 55, 1369–1381. 10.1152/jn.1986.55.6.1369 3734861

[B17] Longo A. Haid T. Meulenbroek R. Federolf P. (2019). Biomechanics in posture space: Properties and relevance of principal accelerations for characterizing movement control. J. Biomech. 82, 397–403. 10.1016/j.jbiomech.2018.11.031 30527635

[B18] Pellegrini B. Zoppirolli C. Boccia G. Bortolan L. Schena F. (2018). Cross-country skiing movement factorization to explore relationships between skiing economy and athletes' skills. Scand. J. Med. Sci. Sports 28, 565–574. 10.1111/sms.12938 28649805

[B19] Promsri A. Haid T. Federolf P. (2018). How does lower limb dominance influence postural control movements during single leg stance? Hum. Mov. Sci. 58, 165–174. 10.1016/j.humov.2018.02.003 29448161

[B20] Promsri A. Haid T. Federolf P. (2020a). Complexity, composition, and control of bipedal postural control system adapts to unstable support surfaces or altered feet positions. Neuroscience 430, 113–124. 10.1016/j.neuroscience.2020.01.031 32027995

[B21] Promsri A. Haid T. Werner I. Federolf P. (2020b). Leg dominance effects on postural control when performing challenging balance exercises. Brain Sci. 10:128. 10.3390/brainsci10030128 32106392 PMC7139434

[B22] Promsri A. Longo A. Haid T. Doix A. C. M. Federolf P. (2019). Leg dominance as a risk factor for lower-limb injuries in downhill skiers—a pilot study into possible mechanisms. Int. J. Environ. Res. Public Health 16:3399. 10.3390/ijerph16183399 31540226 PMC6765833

[B23] Troje N. F. (2002). Decomposing biological motion: a framework for analysis and synthesis of human gait patterns. J. Vis. 2, 371–387. 10.1167/2.5.2 12678652

[B24] Verheul J. Warmenhoven J. Lisboa P. Gregson W. Vanrenterghem J. Robinson M. A. (2019). Identifying generalised segmental acceleration patterns that contribute to ground reaction force features across different running tasks. J. Sci. Med. Sport. 22, 1355–1360. 10.1016/j.jsams.2019.07.006 31445948

[B25] Verrel J. Lövdén M. Schellenbach M. Schaefer S. Lindenberger U. (2009). Interacting effects of cognitive load and adult age on the regularity of whole-body motion during treadmill walking. Psychol. Aging 24, 75–81. 10.1037/a0014272 19290739

[B26] Wachholz F. Kockum T. Haid T. Federolf P. (2019a). Changed temporal structure of neuromuscular control, rather than changed intersegment coordination, explains altered stabilographic regularity after a moderate perturbation of the postural control system. Entropy 21:614 10.3390/e21060614 PMC7515107 33267328

[B27] Wachholz F. Tiribello F. Mohr M. van Andel S. Federolf P. (2020). Adolescent awkwardness : alterations in temporal control characteristics of posture with maturation and the relation to movement exploration. Brain Sci. 10:E216. 10.3390/brainsci10040216 32260555 PMC7226109

[B28] Wachholz F. Tiribello F. Promsri A. Federolf P. (2019b). Should the minimal intervention principle be considered when investigating dual-tasking effects on postural control? Brain Sci. 10:1 10.3390/brainsci10010001 31861521 PMC7016962

[B29] Winter D. A. (1995). Human balance and posture control during standing and walking. Gait Posture 3, 193–214. 10.1016/0966-6362(96)82849-9

[B30] Winter D. A. Sidwall H. G. Hobson D. A. (1974). Measurement and reduction of noise in kinematics of locomotion. J. Biomech. 7, 157–159. 10.1016/0021-9290(74)90056-6 4837552

[B31] Zago M. Codari M. Iaia F. M. Sforza C. (2017a). Multi-segmental movements as a function of experience in karate. J. Sports Sci. 35, 1515–1522. 10.1080/02640414.2016.1223332 27560105

[B32] Zago M. Pacifici I. Lovecchio N. Galli M. Federolf P. A. Sforza C. (2017b). Multi-segmental movement patterns reflect juggling complexity and skill level. Hum. Mov. Sci. 54, 144–153. 10.1016/j.humov.2017.04.013 28499158

[B33] Zago M. Sforza C. Bona A. Cimolin V. Costici P. F. Condoluci C.. (2017c). How multi segmental patterns deviate in spastic diplegia from typical developed. Clin. Biomech. 48, 103–109. 10.1016/j.clinbiomech.2017.07.016 28806590

