# Supplementary material for: Analysis of Postural Control Using Principal Component Analysis: The Relevance of Postural Accelerations and of Their Frequency Dependency for Selecting the Number of Movement Components
Source: Front Bioeng Biotechnol. 2020 May 19;8:480. doi: 10.3389/fbioe.2020.00480 (PMC7248432; doi:10.3389/fbioe.2020.00480)
Supplement: Supplementary file 3 [file Data_Sheet_1.PDF]

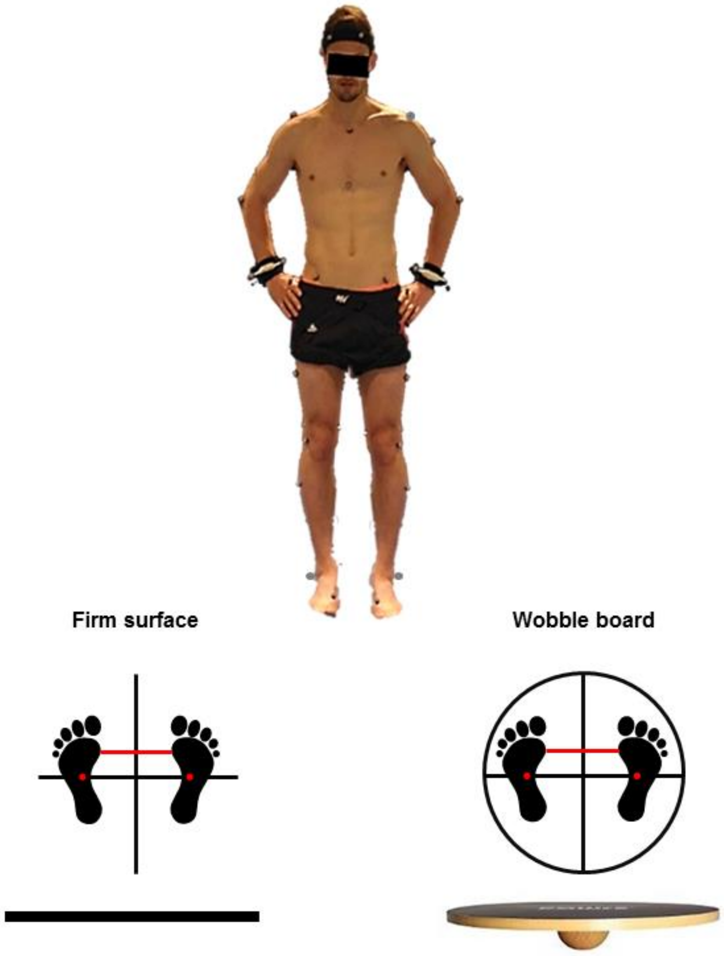

Supplementary Figure 1. Illustration of the starting position and the standardized foot positions for balancing on the firm surface (FS) and on the wobble board (WB). The red marked point on each foot represented the base of each 2nd metatarsal bone and the red horizontal line represented an inter-feet distance (15% of individual biacromial diameter) between the medial borders of each distal end of the first metatarsal bone.

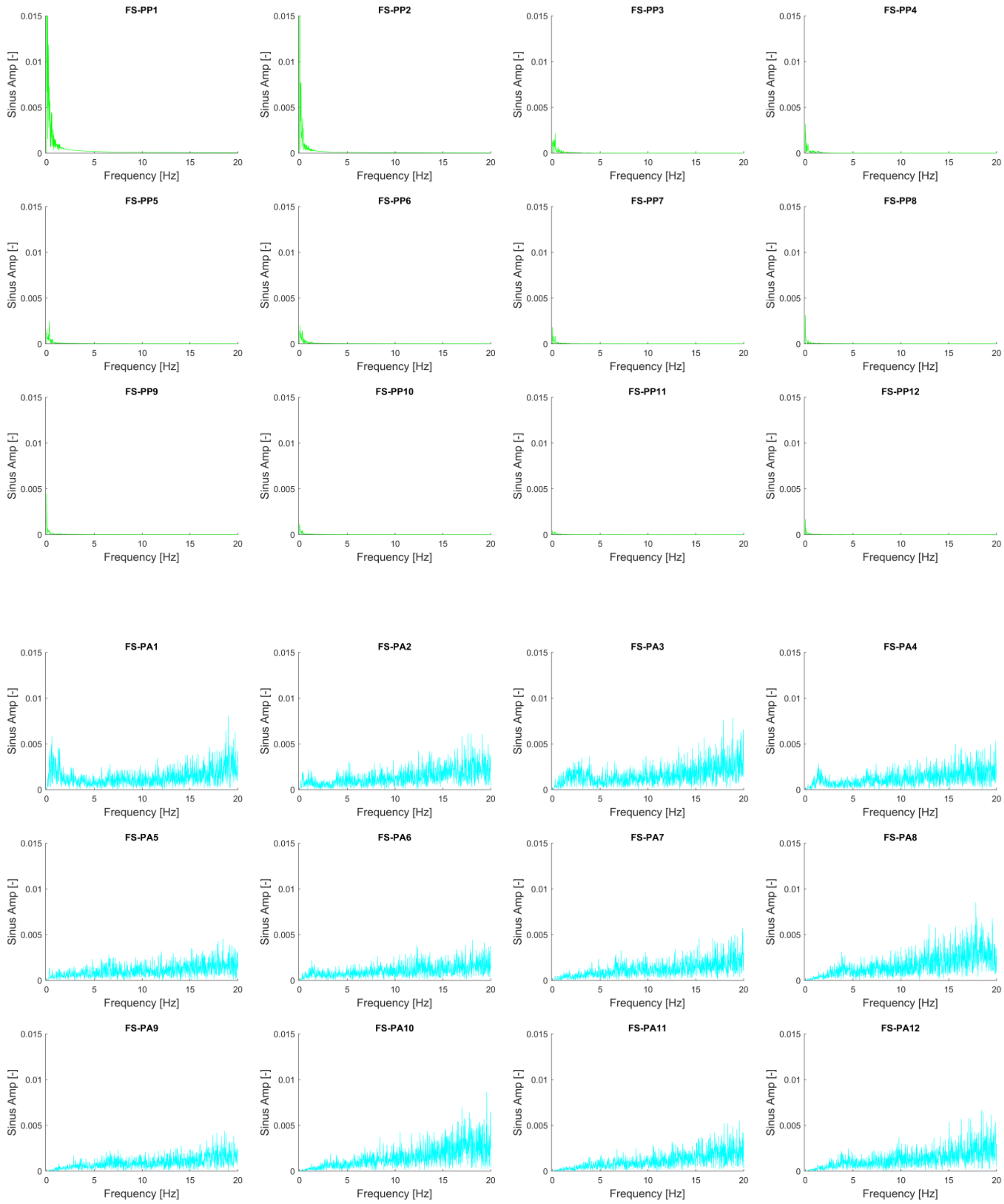

Supplementary Figure 2. Illustration of the frequency content of the first 12 unfiltered principal position (PP_k) and principal acceleration (PA_k) time-series (k orders the order of principal components) of one arbitrarily selected volunteer standing on the firm surface (FS).

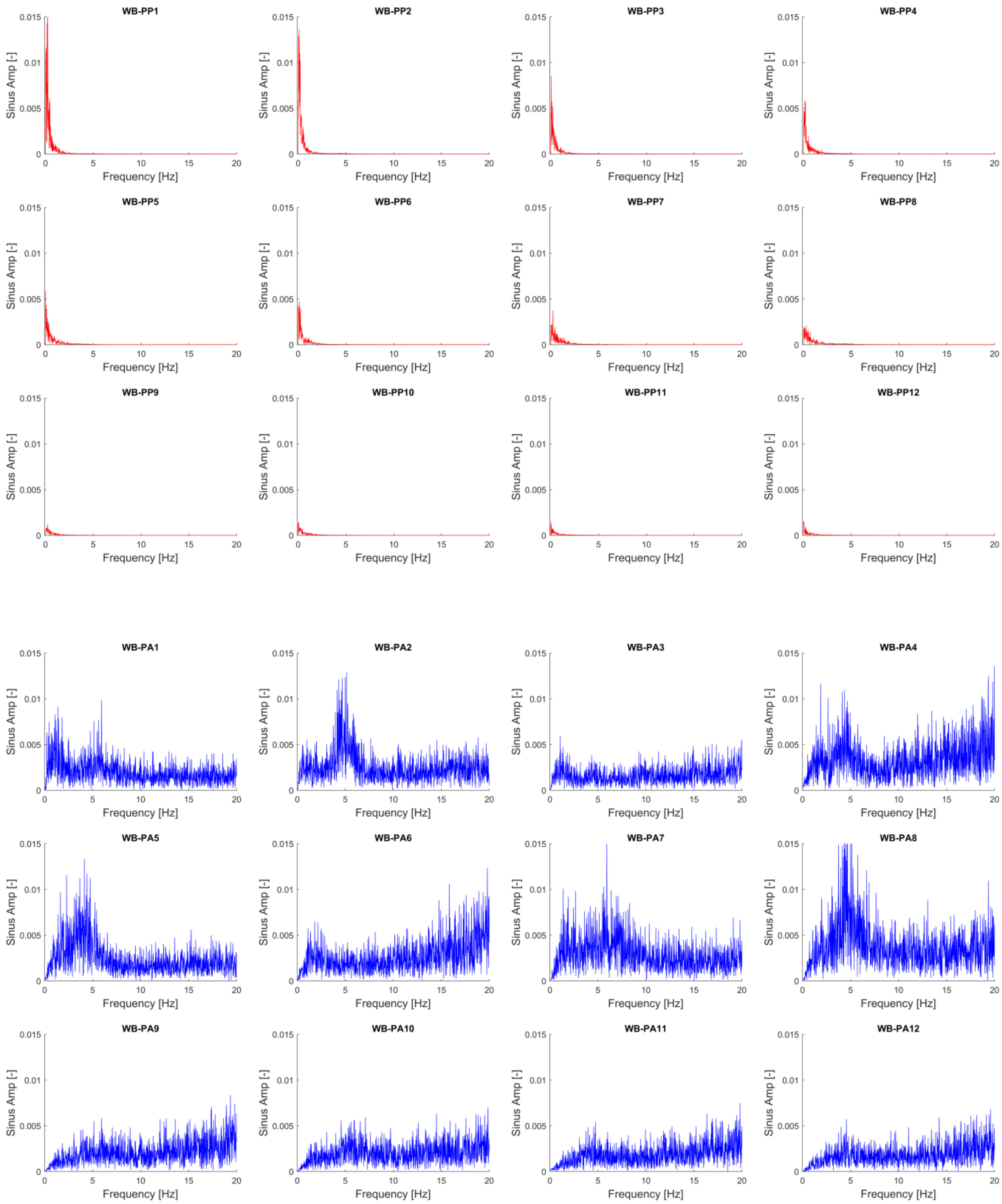

Supplementary Figure 3. Illustration of the frequency content of the first 12 unfiltered principal position (PP_k) and principal acceleration (PA_k) time-series (k orders the order of principal components) of one arbitrarily selected volunteer balancing on the wobble board (WB).
